# C-reactive Protein (CRP) in Patients With Myocarditis: A Systematic Review and Meta-Analysis

**DOI:** 10.7759/cureus.71885

**Published:** 2024-10-19

**Authors:** Bushra Ghulam, Zahira Bashir, Amber Khurshid Akram, Qudsia Umaira Khan, Mamoon Qadir, Shokat Hussain, Amna Akbar, Sarosh Khan Jadoon

**Affiliations:** 1 Biochemistry, Islamic International Medical College, Islamabad, PAK; 2 Biochemistry, Mohi-ud-Din Islamic Medical College, Mirpur, PAK; 3 Biochemistry, Mohi-ud-din Islamic Medical College, Mirpur, PAK; 4 Physiology, Combined Military Hospital Medical College and Institute of Dentistry, Lahore, PAK; 5 Interventional Cardiology, Kulsum International Hospital, Islamabad, PAK; 6 Interventional Cardiology, Polyclinic Hospital Islamabad, Islamabad, PAK; 7 Medicine, Combined Military Hospital, Muzaffarabad, PAK; 8 Emergency and Accident, District Headquarter Hospital, Muzaffarabad, PAK; 9 General Surgery, Sheikh Khalifa Bin Zayed (SKBZ) Combined Military Hospital, Muzaffarabad, PAK

**Keywords:** acute myocarditis, cardiovascular, diagnosis, inflammation, prognosis

## Abstract

Myocarditis is a type of cardiovascular disease related to inflammation of cardiac muscle which can be even fatal to some extent. Early and simple diagnosis is crucial for this complication; however, complex or machine-based methods, such as histological tests, x-rays, electrocardiograms, etc., are usually used for its detection. C-reactive protein (CRP) is a biomarker that naturally elevates during inflammation. Therefore, we tried to understand the correlation between CRP and myocarditis.

We primarily identified 451 studies from PubMed, Google Scholar, and ScienceDirect and ultimately selected four studies as eligible. We identified the mean difference (MD) in CRP levels between the myocarditis patients and healthy controls. The study quality, outliers, sensitivity, significance, and heterogeneity were also checked.

The MD (6.03 (95%CI: 2.41-9.64), p<0.00001) corresponds to a higher and significant CRP level in myocarditis as compared to the control group. The study quality was found to be high with no bias or outliers and the heterogeneity was also determined to be high (I^2^=99%). Using the fixed effect model, the forest plot determined a similar result as the main outcome (MD: 5.08 (95%CI: 4.85-5.32)) proving higher sensitivity and reproducibility. These findings indicated the possibility of CRP being an established biomarker for an accurate diagnosis and prognosis of myocarditis.

## Introduction and background

Myocarditis, also known as the inflammation of cardiac muscle, particularly the middle layer of the heart wall known as the myocardium weakens the cardiac tissue and affects the cardiac electrical system leading to cardiomyopathy and gradual dysfunction of the heart which may eventually progress to cardiac arrest and in the worst cases the development of heart failure [1,2]. Myocarditis is characterized by heterogeneous clinical manifestations including chest discomfort and pain, palpitations, irregular heartbeats, cardiac arrhythmias, increased fatigability, organ swelling, muscle and joint aches, dizziness, etc. Symptoms may be acute to chronic and if left unattended, complications may occur in some patients including cardiomyopathy, cardiogenic shock, lung problems, cardiac failure, and sudden cardiac death [3-5].

Myocarditis is usually classified into four distinct classes, e.g., acute, fulminant, chronic active, or chronic persistent based on the disease manifestation period and types [6]. Myocarditis is often caused by getting infected with a wide variety of microorganisms, e.g., viruses, bacteria, parasites, and fungi, or the administration of certain medicines and drugs, exposure to certain chemicals, toxins, and radiation, or some health conditions including systemic lupus, sarcoidosis, inflammatory bowel diseases, giant cell arteritis, granulomatosis, Takayasu's arteritis, acute rheumatic fever, etc. [7-10]. Despite being a rare cardiac disease, the current global prevalence of myocarditis ranges from 10.2 to 105.6 per 100,000 with an incidence of 1.8 million new cases annually [11]. Supportive and multispecialty treatment approaches are often recommended for myocarditis patients to prevent progression and complications. Conventional treatment procedures include administering analgesic and anti-inflammatory medication, different non-steroidal anti-inflammatory drugs to reduce inflammation and further damage, corticosteroids to decrease immune activity, particularly the autoimmune responses, intravenous administration of immunoglobulins to maintain balanced immune responses, and so on. In cases of severe complications and advanced stages of myocarditis, different surgical processes, and mechanical circulatory support devices are required [4,12].

Diagnosis of myocarditis includes physical examination and different tests including histological tests, chest x-ray, electrocardiogram (ECG), cardiac MRI, positron emission tomography (PET), cardiac catheterization, endomyocardial biopsy, etc. [12-14]. These current techniques for the diagnosis of myocarditis are often expensive, time-consuming, and invasive indicating the necessity of a more efficient, less expensive, and accurate method for earlier screening, establishing a diagnosis, and determining the prognosis of myocarditis. Blood enzyme tests for the detection of myocarditis-specific biomarkers hold the potential to be a good alternative to conventional diagnostic approaches [15]. Among the biomarkers studied so far, several myocardial necrosis and circulating biomarkers, e.g., cardiac troponin (cTn), natriuretic peptides, creatinine kinase (CK), creatinine kinase-MB (Ck-MB), angiotensin-converting enzymes (ACE), autoantibodies, eosinophilic cationic protein (ECP), and different genetic biomarkers particularly the microRNA and some non-coding RNA are well commonly used for the diagnosis and prognosis of myocarditis [15,16]. However, these biomarkers are also related to the diagnosis of other diseases such as cTn is particularly related to Myocardial infarction (MI), Ck-MB to cardiac ischemia and liver failure, natriuretic peptides to heart failure (HF), ACE to kidney and renal diseases, autoantibodies to autoimmunity and autoimmune related cardiovascular diseases (CVD), ECP to asthma, and microRNA and some non-coding RNA to cancer [17-22].

C-reactive protein (CRP), a prototype inflammatory biomarker is one of the most extensively studied and widely used prognostic biomarkers which is mainly produced in the liver by the hepatocytes or immune cells such as lymphocytes or macrophages [23,24]. Production of CRP can be increased up to a thousandfold in response to inflammation and tissue necrosis by inflammatory cytokines such as interleukin-1 and tumor necrosis factor [16]. The difference between x-ray, ECG, MRI, PET, histopathology, biopsy, and such conventional tests and CRP assessment is that the conventional tests are mostly mapping and/or imaging-based whereas CRP helps to determine the serological condition and thus assess the cellular damage, inflammation, and the criticality of a myocarditis patient [25,26]. Although CRP was reported to be a potential biomarker to detect inflammation which is correlated as an important symptom of myocarditis, no investigation has been done to date to identify the association of CRP with myocarditis. Therefore, this systematic review and meta-analysis aims to determine the association between the presence of CRP and myocarditis.

## Review

Methodology

Study Guidelines, Search Techniques, and Inclusion

This study was conducted following the PRISMA guideline obtained from the previous studies [27,28]. Three different databases, i.e., PubMed, ScienceDirect and Google Scholar were searched with specific keywords such as “C-reactive protein,” “CRP,” and “myocarditis” with certain search techniques. In PubMed, the “advanced” search was done using “title and abstract” in the query field whereas in ScienceDirect “title, abstract, keywords” of the advanced search option was used. In Google Scholar, the same key terms were used by utilizing the “allintitle” option in the search bar. The Boolean operators were used where required. The search technique was obtained from previous studies with some slight modifications [29,30]. The search was conducted and completed in September 2024. After the search was completed, the authors rigorously searched for research articles that had data regarding CRP levels in patients with myocarditis which was compared with healthy controlled patients. All the other articles such as comprehensive reviews, systematic reviews, case reports, letters to the editor, correspondence, and editorials were excluded from this study.

Characteristics and Quality of the Studies

This meta-analysis used the mean value and standard deviation (SD) of the CRP within the study participants along with the total number of participants of two comparative groups including myocarditis patients and healthy control groups, respectively. Moreover, to characterize all the selected studies; the data of study type, study location, study period, study settings, and participants' demographics including the percentage of male and female participants with the mean age in years, types of myocarditis, and the methods used for CRP detection were extracted from the selected studies. The quality of the selected studies was investigated by finding the answers to the quality-checking questions from NIH and UNC [31,32]. For a particular question, if the answer was found and stated clearly (Y), the study got one mark, if not found (N) as it was not done in that study, a zero mark was used, if the answer was partially (P) reported in the study then it got 0.5 mark, and if not reported (NR) then it got no mark. Ultimately for eight different questions, the total obtained points were converted into percentages to estimate the overall score. Based on the overall score, the quality of the selected studies was investigated following previous studies with slight modifications [33]. The percentage score >70% was regarded as high-quality (low bias risk) and <50% was regarded as low-quality (high bias risk). The score in-between was regarded as moderate quality (moderate bias risk).

Bias, Meta, and Sensitivity Investigation 

Study bias was investigated to observe the asymmetry and presence of outlier studies among the selected studies. This was investigated using a funnel plot following a previous study [34]. To determine the heterogeneity, the I^2^ value was used. Here, I^2^>75% was regarded as significant heterogeneity following a previous study [33]. Heterogeneity indicates the variation in the study outcomes among different studies [35]. The meta-analysis with the main extracted data of the included studies was also done using a forest plot with a random effect model. The main target was identifying the odd ratio (OR) and the 95% confidence interval (95% CI) through the forest plot. Another forest plot was created using a fixed effect model to observe the reproducibility and sensitivity of the main forest plot [33]. All these analyses were done using RevMan software (version 5.4; Cochrane, London). 

Subgroups

Based on the different types of myocarditis, a subgroup analysis was done using the forest plot with a random effect model.

Results

Study Selection and Characterization

**Figure 1:**
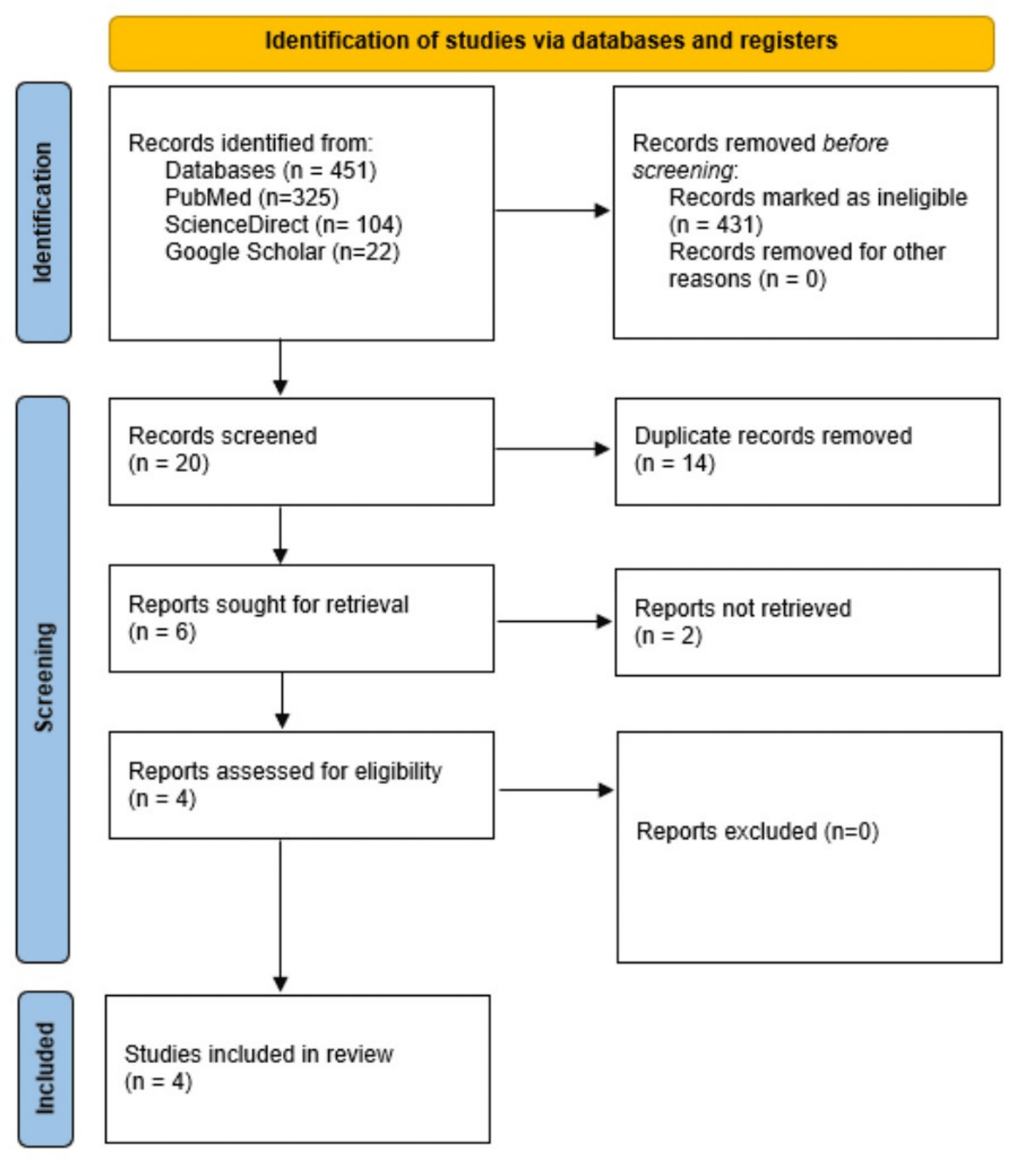
PRISMA diagram of the study inclusion strategy. Primarily 451 articles were found after applying the specific search strategy in PubMed (n=325), ScienceDirect (n=104), and Google Scholar (n=22). Four hundred thirty-one articles were primarily excluded due to the ineligibility of not being full-length research articles. Of the remaining 20 articles, 14 were excluded due to study duplication and two were excluded due to the topic's irrelevance and data unavailability. Finally, four articles were selected and included in this study.

The search strategy identified a total of 451 articles from three databases: PubMed (n=325), ScienceDirect (n=104), and Google Scholar (n=22). Initially, 431 articles were excluded as they did not meet the criteria of being full-length research articles. Of the remaining 20 articles, 14 were removed due to duplication, and two were discarded for lacking relevance to the studies of interest as well as lacking data availability, although the quality of the excluded studies was also decent, and they were peer-reviewed published articles. Ultimately, four articles were chosen for inclusion in this study [36-39]. Figure 1 illustrates the step-by-step search and selection process using the PRISMA flow chart.

We thoroughly evaluated and extracted the data variables for the study characteristics from the four selected articles. Table 1 provides a comprehensive breakdown of the data variables from these selected studies. 

**Table 1:** Characteristics table Here, Y= Years, NR= Not reported, CRP= C reactive protein, ELISA= Enzyme linked immunosorbent assay

Study ID	Study type	Location	Study period	Study settings	Patient demographic			Myocarditis type	CRP detection method	Reference
					Male, (%)	Female, (%)	Mean Age (Y)			
Chen J et al. 2019	Case-control	China	Mar 2015-Feb 2017	Third Xiangya Hospi- tal	59.57	40.42	5.25	Viral	Immunoturbidimetry assay	[26]
Gao W et al. 2023	Case-control	China	Jan 2020-April 2022	Si County People's Hospital	NR	NR	NR	Infective	NR	[27]
Kaneko K et al. 2000	Case-control	Japan	1988-1997	Gunma University Hospital	54.83	45.16	38.30	Lymphocytic	NR	[28]
Qin Q et al. 2020	Case-control	China	Jun 2017- Jun 2019	The Affiliated Hospital of Xuzhou Medical University	59.35	40.65	58.12	Viral	ELISA	[29]

Quality, Outlier, and Heterogeneity

The quality of all the selected studies was of high quality (scored >70%) with two studies obtaining 100%, one study obtaining an 87.5% score, and one study obtaining 75% (Table 2).

**Table 2:** Quality investigation of the included studies 1. Was the research question appropriate? 2. Was the study population clearly defined? 3. Were cases and controls selected from a similar population? 4. Was a proper timeframe maintained? 5. Did the author follow proper inclusion and exclusion criteria regarding case-control selection? 6. Were the cases clearly defined and differentiated from controls? 7. Were the methods of quantity determination clearly defined? 8. Did the authors use statistical analyses? 9. Were the measurements/tools valid to conduct the study?  Y=Yes (Score=1), N=No (Score=0), P= Partially (Score=0.5), NR= Not reported (No score)

Study ID	1	2	3	4	5	6	7	8	Overall (%)
Chen J et al. 2019 [26]	Y	Y	Y	Y	Y	Y	Y	Y	100
Gao W et al. 2023 [27]	Y	Y	Y	Y	NR	Y	NR	Y	75
Kaneko K et al 2000 [28]	Y	Y	Y	Y	Y	Y	N	Y	87.5
Qin Q et al. 2020 [29]	Y	Y	Y	Y	Y	Y	Y	Y	100

**Figure 2:**
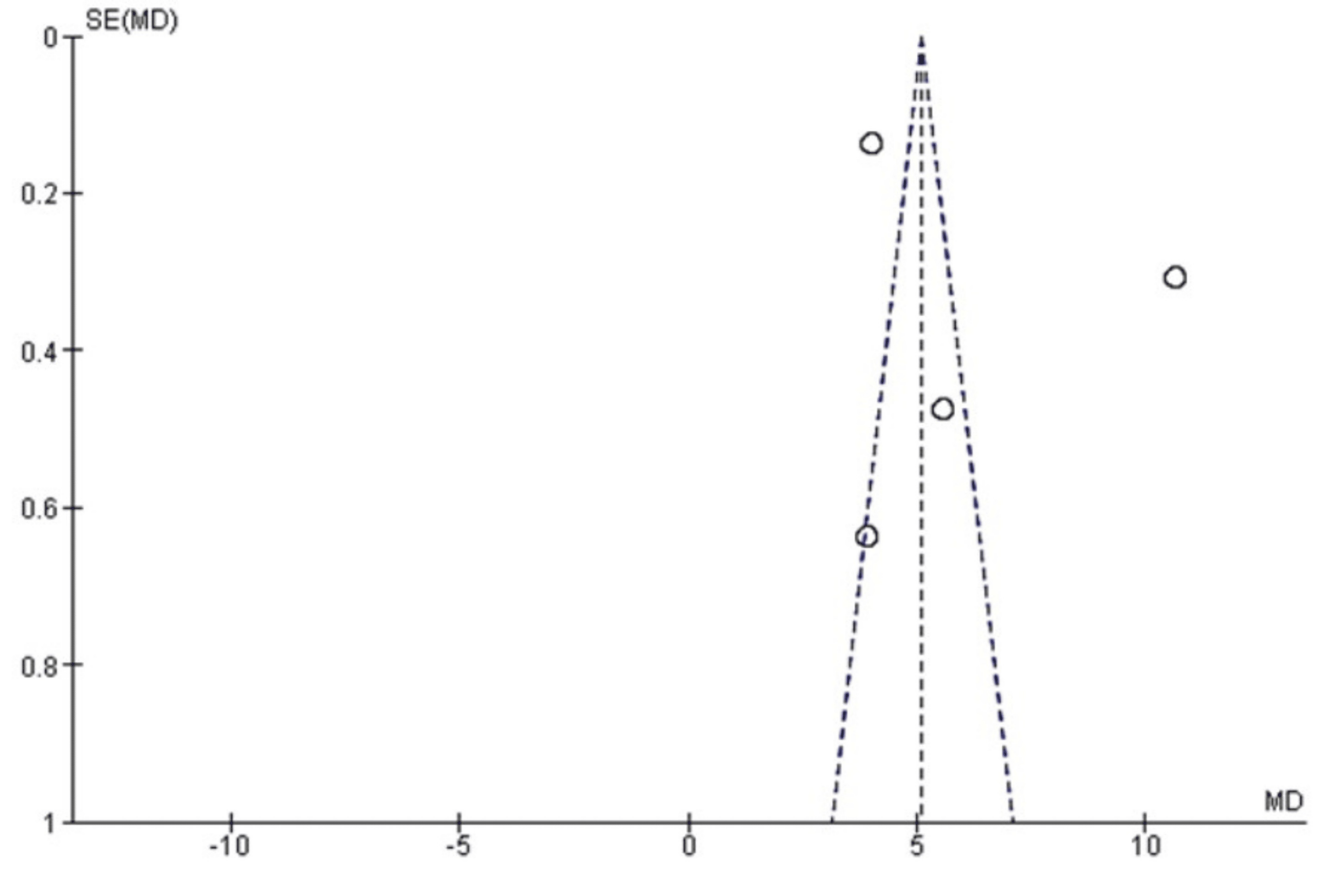
Funnel plot detecting outlier studies. The funnel plot found no significant outliers.

This further indicated that the studies were of low bias risk. Furthermore, the study bias assessment through the funnel was also measured; however, the plot did not identify any significant study asymmetry or outliers (Figure 2).

That reconfirmed that all our included studies were symmetrical without any major asymmetry for the meta-analysis. Also, the heterogeneity of the selected studies was identified to be significant (I^2^=99%).

Meta and Sensitivity

**Figure 3:**
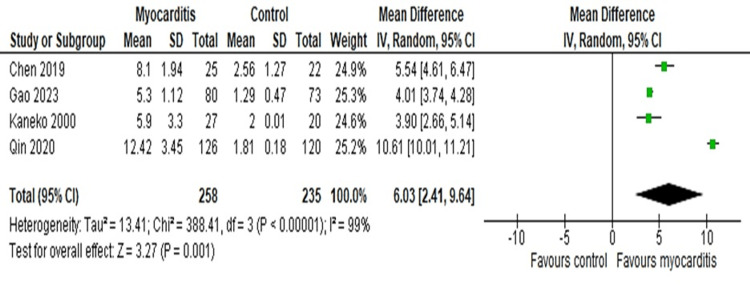
Forest plot of the main outcome Forest plot using random effect model detecting that the CRP is higher in concentration within myocarditis patients than in the control group [26-29].

The main forest plot using the random effect model detected the value of the mean difference (MD) as 6.03 (95%CI: 10.01-11.21) which indicated that the CRP was higher in myocarditis patients as compared to healthy control patients (Figure 3).

**Figure 4:**
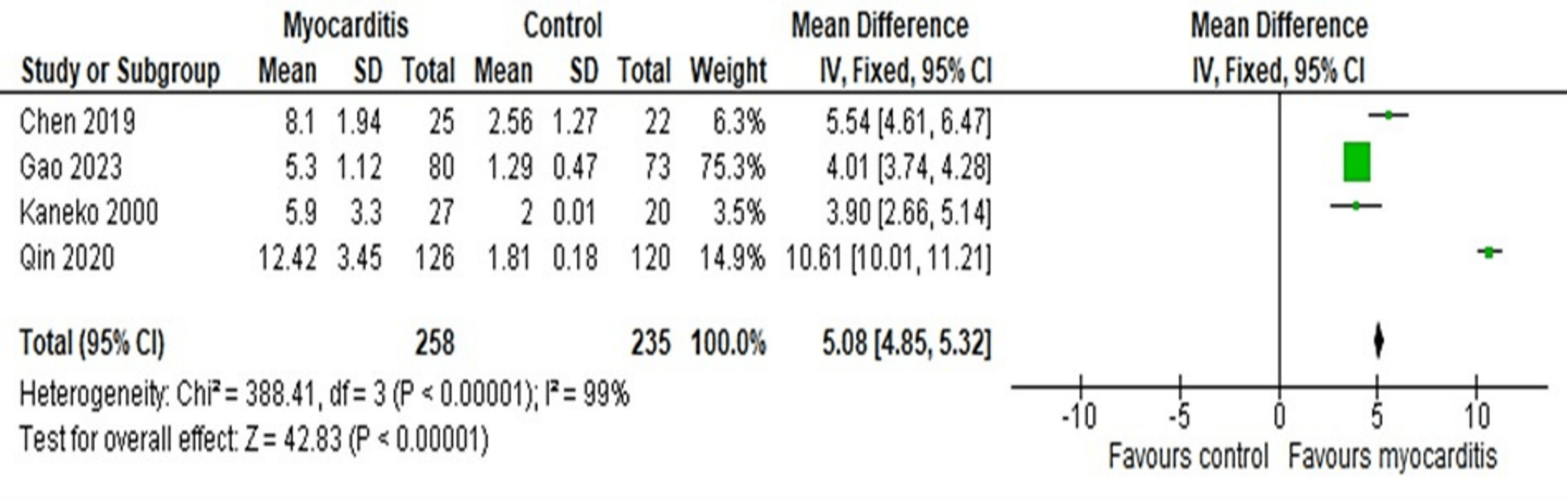
Forest plot to determine the sensitivity and reproducibility of the main outcome. Forest plot using the fixed effect model further detects that the CRP is higher in concentration within myocarditis patients than in the control group [26-29].

However, for the reassessment of the sensitivity and reproducibility of the main meta-outcome, a fixed effect model was used to regenerate the same forest plot. As a result, MD was determined as 5.08 (95%CI: 4.85-5.32) which further implied our main analysis to be correct (Figure 4).

Subgroup-Based Findings

**Figure 5:**
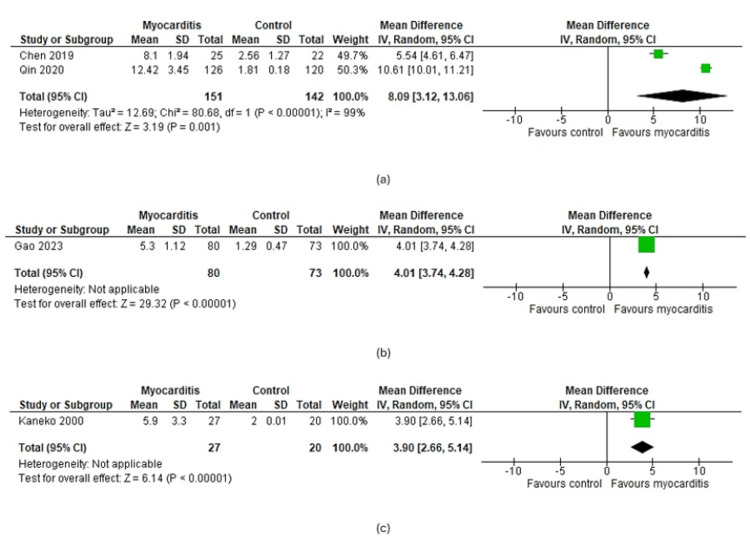
Forest plots of subgroup analyses. Forest plot detecting that CRP is higher in concentration within myocarditis patients than in the control group in (a) viral, (b) infective, and (c) lymphocytic myocarditis [26-29].

The subgroup analysis was done based on 3 types of myocarditis such as viral, infective, and lymphocytic myocarditis. As a result, the MD was found to be the highest in viral myocarditis (8.09 (95%CI: 3.12-13.06)), followed by infective (4.01 (95%CI: 3.74-4.28)) and lymphocytic (3.09 (95%CI: 2.66-5.14)) myocarditis (Figure 5) 

Discussion

The present systematic review and meta-analysis were performed to get insight into the association between the presence and magnitude of CRP in patients suffering from myocarditis as compared to the control participants. To the best of the author’s knowledge, this is the first meta-analysis to determine the association between CRP and myocarditis based on the findings of the available research articles found during literature screening. CRP is an acute phase reaction protein, synthesized by the hepatocytes during stress conditions, particularly during infections and subsequent tissue damage or necrosis which induce the release of proinflammatory cytokines, e.g., IL-1, IL-6, and TNF-alpha [40,41]. CRP remains in a very low concentration during a healthy period but in cases of inflammation and tissue damage, its production increases notably in response to the inflammatory cytokines [42]. CRP induces the initiation of immune responses through complement system activation and enhancing the ability of the professional phagocytes, e.g., macrophages, eosinophils, and neutrophils to engulf the infectious agents or the necrotic tissues and apoptosis of the responsible cells [23]. During myocarditis, the cardiac cells lying in the middle layer of the cardiac wall get damaged which eventually leads to the release of inflammatory cytokines resulting in the upregulation of CRP synthesis and heightened immune responses against the causative agents. Thus, CRP along with some other biomarkers with similar activities including TNF-alpha, troponin, and creatinine kinase are frequently used as diagnostic and prognostic markers of myocarditis along with the determination of its severity and progressive stages [15].

Though the MD value was determined by the meta-analysis and construction of a forest plot in a random effects model, it was found that CRP was significantly higher among the myocarditis patients (6.03; 95% CI: 2.41-9.64) compared to the control participants indicating increased production and persistence of CRP in response to the immune activation due to cardiac cellular damage and inflammation (Figure 3). Among the four included studies, Qin 2020 showed a notably higher level of CRP among myocarditis patients (MD: 10.61; 95% CI: 10.01-11.21) compared to the other studies since this study included patients suffering from viral myocarditis whereas the other studies centered on infective or lymphocytic myocarditis (Table 1, Figure 3) [36-39].

The subgroup analysis of this study determined significantly higher levels of CRP among the viral myocarditis patients (8.09; 95% CI: 3.12-13.06) compared to the infective (4.01; 95% CI: 3.74-4.28) and lymphocytic myocarditis (3.90; 95% CI: 2.66-5.14) (Figure 5). A wide array of viruses including hepatitis B and C virus, Epstein-Barr virus, echovirus, parvovirus, Coxsackie virus, Ekage virus, enterovirus, and adenovirus including influenza, parainfluenza, and COVID-19 can cause increased synthesis of CRP by directly damaging the cardiomyocytes and releasing antigens that eventually activates the heightened immune responses [43,44]. Higher levels of CRP found among the patients of these two studies occurred since these studies included participants with viral infections, particularly Chen which included myocarditis patients with a previous history of respiratory tract infections with Coxsackie and adenovirus infections among some of the participants [36,39]. SARS-CoV-2, belonging to the adenovirus can also cause damage to the cardiomyocytes leading to the development of myocarditis and increased levels of CRP which can be tested as a potential biomarker as stated by Srivastava et al. [45]. Besides myocardial damage, viral infections often cause inflammation and tissue injury of other organs, particularly the respiratory organs leading to the upregulation of CRP production which can also contribute to higher levels of CRP among viral myocarditis patients [46]. On the contrary, lymphocytic and infective myocarditis are usually caused due to the damage of cardiac cells by lymphocytes which were produced as an immune response against different microbes, particularly viruses, as well as after infections [47]. As the infections heal, CRP production is downregulated, and the level decreases which may again increase to some extent during myocardial inflammation caused by the lymphocytes which indicates the probable reason behind lower CRP levels among the infective and lymphocytic myocarditis patients compared to the viral myocarditis. Moreover, the baseline level of CRP increases with aging and is usually higher among older individuals compared to children and middle-aged persons [48]. Among the included studies, Qin included viral myocarditis patients with a mean age of 58.12 years which might also contribute to the higher CRP levels found among the viral myocarditis subgroup compared to the other subgroups with lower-aged participants [39].

A higher degree of heterogeneity (99%) was found in the random and fixed effects model along with the subgroup analysis with the viral myocarditis patients which indicates substantial variations among the included studies making it difficult to generalize the meta-analysis outcomes to the entire study population (Figures 3-5). This kind of variation may result from several factors including variation in study types, study duration and study setting, sample size, detection and confirmation methods, etc. [49]. However, the main plausible cause of the high heterogeneity of our analyses is the variation in the mean data and the number of patients.

Due to its strong association with myocarditis, CRP levels are tested as a routine histological diagnosis assay and are widely used for earlier detection and monitoring of myocarditis [50]. Besides, CRP levels were also found to be associated with other CVD such as coronary artery disease (CAD), HF, acute coronary syndrome (ACS), and arterial fibrillation (AF) as well in previous studies [51-54]. However, CRP acts as a non-specific diagnostic biomarker for accurate diagnosis of myocarditis [55]. Therefore, the standalone value of CRP levels is limited as it is primarily a marker of inflammation and does not accurately differentiate the source of inflammation. Application of first-line tests, e.g., electrocardiography, nuclear imaging, cardiovascular magnetic resonance (CMR) imaging, chest x-ray, and in some instances the second-line diagnostic approaches, e.g., cardiac catheterization and endomyocardial biopsy are recommended with routine tests for biomarkers in patients with clinical manifestations of myocarditis [6,56]. Our findings strongly suggest that CRP can be a strong biomarker for the diagnosis and prognosis of myocarditis especially when merged with the other conventional methods.

Limitations of the study

We could find a small number of studies that are eligible for this systematic review and meta-analysis. Besides, the heterogeneity of the included studies was found to be high.

## Conclusions

We found a strong association between the enhanced levels of CRP and myocarditis as compared to healthy control patients. Therefore, CRP needs to be considered as a potential diagnostic and prognostic biomarker for myocarditis. Primarily, it can be considered a first-line biomarker besides the conventional and mechanical tests until it is established as a confirmatory biomarker of myocarditis. More research is required to obtain relevant data regarding its association with myocarditis to further assess its sensitivity, specificity, and accuracy.

## Appendices

**Table 3:** Supplementary search techniques

Database	Search techniques
PubMed	(myocarditis[Title/Abstract]) AND ((crp[Title/Abstract]) OR (c reactive protein[Title/Abstract]))
ScienceDirect	Title, abstract, keywords: C reactive protein myocarditis Title, abstract, keywords: CRP myocarditis
Google Scholar	allintitle: CRP myocarditis allintitle: c reactive protein myocarditis
